# Phase 2 Study of Anti-Human Cytomegalovirus Monoclonal Antibodies for Prophylaxis in Hematopoietic Cell Transplantation

**DOI:** 10.1128/AAC.02467-19

**Published:** 2020-03-24

**Authors:** Johan Maertens, Aaron C. Logan, Junho Jang, Gwynn Long, Jih-Luh Tang, William Y. K. Hwang, Liang Piu Koh, Roy Chemaly, Armin Gerbitz, Julia Winkler, Su-Peng Yeh, John Hiemenz, Sandra Christoph, Dong-Gun Lee, Po-Nan Wang, Ernst Holler, Stephan Mielke, Luke Akard, Adeline Yeo, Sangana Ramachandra, Kristin Smith, Peter Pertel, Florencia Segal

**Affiliations:** a Universitaire Ziekenhuizen Leuven, Leuven, Belgium; b University of California, San Francisco Medical Center, San Francisco, California, USA; c Samsung Medical Center, Seoul, South Korea; d Duke University Medical Center, Durham, North Carolina, USA; e National Taiwan University, Taipei, Taiwan; f Singapore General Hospital, Singapore; g National University Cancer Institute Singapore, Singapore; h University of Texas Anderson Cancer Center, Houston, Texas, USA; i Universitatsklinikum Erlangen, Erlangen, Germany; j China Medical University Hospital, Taichung, Taiwan; k University of Florida Health Shands Cancer Hospital, Gainesville, Florida, USA; l Universitatsklinikum Essen, Essen, Germany; m Seoul St. Mary’s Hospital, Seoul, South Korea; n Chuang Gung Memorial Hospital—Linkou Branch, Linkou, Taiwan; o Universitatsklinikum Regensburg, Regensburg, Germany; p Universitatsklinikum Wurzburg, Wurzburg, Germany; q Indiana Blood and Marrow Transplantation Clinic, Indianapolis, Indiana, USA; r Stat4ward LLC, Pittsburgh, Pennsylvania, USA; s USA Novartis Institutes for BioMedical Research, Cambridge, Massachusetts, USA

**Keywords:** hematopoietic stem cell transplantation, human cytomegalovirus, prophylaxis

## Abstract

Human cytomegalovirus (HCMV) can cause significant disease in immunocompromised patients, and treatment options are limited by toxicities. CSJ148 is a combination of two anti-HCMV human monoclonal antibodies (LJP538 and LJP539) that bind to and inhibit the functions of viral HCMV glycoprotein B (gB) and the pentameric complex, consisting of glycoproteins gH, gL, UL128, UL130, and UL131. In this phase 2, randomized, placebo-controlled trial, we evaluated the safety and efficacy of CSJ148 for prophylaxis of HCMV in patients undergoing allogeneic hematopoietic stem cell transplantation. As would be expected in the study population, all the patients (100%) reported at least one treatment-emergent adverse event. There were 22 deaths during this study, and over 80% of the patients receiving placebo or CSJ148 developed at least one adverse event of grade 3 or higher severity. No subject who received antibody developed a hypersensitivity- or infusion-related reaction. CSJ148-treated patients showed trends toward decreased viral load, shorter median duration of preemptive therapy, and fewer courses of preemptive therapy. However, the estimated probability that CSJ148 decreases the need for preemptive therapy compared to placebo was 69%, with a risk ratio of 0.89 and a 90% credible interval of 0.61 to 1.31. The primary efficacy endpoint was therefore not met, indicating that CSJ148 did not prevent clinically significant HCMV reactivation in recipients of allogeneic hematopoietic cell transplants. (This study has been registered at ClinicalTrials.gov under identifier NCT02268526 and at EudraCT under number 2017-002047-15.)

## Abstract (precis)

Human cytomegalovirus (HCMV) can cause significant disease in immunocompromised patients, and treatment options are limited by toxicities. CSJ148 is a combination of two anti-HCMV human monoclonal antibodies (LJP538 and LJP539) that bind to and inhibit the functions of viral HCMV glycoprotein B (gB) and the pentameric complex, consisting of glycoproteins gH, gL, UL128, UL130, and UL131. In this phase 2, randomized, placebo-controlled trial, we evaluated the safety and efficacy of CSJ148 for prophylaxis of HCMV in patients undergoing allogeneic hematopoietic stem cell transplantation.

## INTRODUCTION

Human cytomegalovirus (HCMV) infection is common, with approximately 60% of the population worldwide infected (1). Most infections are mild or asymptomatic, but clinically significant HCMV disease and complications can occur in immunocompromised individuals. These individuals include hematopoietic cell transplant (HCT) and solid organ transplant recipients, people infected with human immunodeficiency virus (HIV), and newborns exposed to HCMV *in utero*. Everyone previously infected with HCMV is at risk for HCMV reactivation and, if immunocompromised, significant disease.

Among HCT recipients, HCMV infection is associated with increased morbidity and mortality, even in the preemptive therapy era (2). Pneumonia is the most serious HCMV disease among HCT recipients, with mortality exceeding 50% (3). Other HCMV manifestations include gastroenteritis, hepatitis, retinitis, and encephalitis (4). In addition, HCMV infection and its treatment are associated with (secondary) graft failure, increased incidence of bacterial and fungal infections, and potentially increased incidence of graft-versus-host disease (GVHD). In the absence of prophylaxis, the majority of HCMV-seropositive allogeneic HCT recipients develop HCMV reactivation after transplantation, and approximately 10 to 15% develop HCMV disease if preemptive therapy is not given (2).

HCMV antivirals include ganciclovir, valganciclovir, foscarnet, and cidofovir. These drugs are effective at preventing and treating HCMV reactivation and disease but are associated with significant toxicities, such as renal toxicity and electrolyte disturbances. Bone marrow toxicity limits the use of these agents for prophylaxis in patients undergoing HCT until after engraftment (4), and up to 15% of patients develop significant HCMV infection and disease during this preengraftment period (5–7). Letermovir, an HCMV-specific terminase complex inhibitor with an improved safety profile, has recently become available for prevention of HCMV infection after HCT (8) and can be used safely during the early preengraftment period. However, letermovir is associated with patient compliance issues, drug-drug interactions, low-grade adverse events, and breakthrough HCMV infections.

CSJ148 consists of two anti-HCMV human monoclonal antibodies (LJP538 and LJP539) (9) and offers the potential to be a safe and well-tolerated alternative to currently available therapies for the prevention and treatment of HCMV. Each antibody binds to and inhibits the function of essential viral glycoproteins; LJP538 binds to glycoprotein B (gB), and LJP539 binds to the pentameric complex (consisting of glycoproteins gH, gL, UL128, UL130, and UL131). CSJ148 neutralizes HCMV infection of all the cell types tested by blocking both initial infection and the subsequent cell-to-cell spread of virus (9).

In healthy volunteers, CSJ148 is safe and well tolerated (10). Adverse events and laboratory abnormalities occurred sporadically, with similar incidences between antibody and placebo groups and without any apparent relationship to dose. After intravenous administration, both LJP538 and LJP539 demonstrated typical human IgG1 pharmacokinetic properties, with slow clearances, limited volumes of distribution, and long terminal half-lives (10). Based on its safety profile, CSJ148 has the potential to be used prior to engraftment to prevent HCMV infection and disease.

The aim of this phase 2 study (ClinicalTrials.gov no. NCT02268526; EudraCT no. 2017-002047-15) was to assess the clinical safety and efficacy of CSJ148 in preventing HCMV infection in HCT recipients during the first 98 days after transplantation.

## RESULTS

### Patients.

Six patients were enrolled in cohort 1, and all received the planned four doses of CSJ148 (Table 1); five completed the study. In cohort 2, 80 patients were enrolled, and 54 patients (38 in the CSJ148-treated group and 16 in the placebo-treated group) completed the study. The most common reasons for discontinuation were death (12; 14.0%), subject’s decision (10; 11.6%), and adverse event (3; 3.5%). The most common reasons for exclusion from the pharmacodynamics (PD) analysis sets were use of medication with anti-HCMV activity prior to meeting a primary endpoint, withdrawal from the study or death prior to meeting the primary endpoint or day 99 (whichever came first), and missing a study drug dose prior to meeting the primary endpoint (see the supplemental material).

**TABLE 1:** Patient disposition

Parameter	Value [no. (%)]			
	Cohort 1, CSJ148 (*n* = 6)	Cohort 2		Total CSJ148 (*n* = 65)
		CSJ148 (*n* = 59)	Placebo (*n* = 21	
Patients				
Completed	5 (83.3)	38 (64.4)	16 (76.2)	43 (66.2)
Discontinued	1 (16.7)	21 (35.6)	5 (23.8)	22 (33.8)
Main cause of discontinuation				
Adverse event(s)	0 (0.0)	2 (3.4)	1 (4.8)	2 (3.1)
Death	1 (16.7)	11 (18.6)	0 (0.0)	12 (18.5)
Protocol deviation	0 (0.0)	1 (1.7)	0 (0.0)	1 (1.5)
Physician's decision	0 (0.0)	0 (0.0)	1 (4.8)	0 (0.0)
Subject’s decision	0 (0.0)	7 (11.9)	3 (14.3)	7 (10.8)
Analysis sets				
Safety and full analysis	6	59	21	65
PK	6	59		65
PD		42	17	42
Combined PD (cohorts 1 and 2)	5	42	17	47
Modified PD (cohort 2 only)		36	16	36
Combined modified PD (cohorts 1 and 2)	5	36	16	41

Baseline demographics and other characteristics were generally comparable between CSJ148-treated and placebo-treated patients (Table 2). All the patients were HCMV seropositive before transplantation. However, the median age of CSJ148-treated patients (56 years) was greater than that of the placebo-treated patients (44 years). In addition, the CSJ148-treated patients were predominantly white, while the placebo-treated patients were predominantly Asian. Based on differences of at least 10%, potentially relevant differences in baseline transplant characteristics included more CSJ148-treated patients receiving a graft from unrelated donors or from HCMV-seronegative donors and a marrow graft, while more placebo-treated patients received T cell-depleted grafts and peripheral blood stem cells. The majority of patients received a graft from a complete-match donor (80% and 71.4% in CSJ148-treated and placebo-treated patients, respectively); haploidentical donors were used in 23.8% of CSJ148-treated and 15.4% of placebo-treated patients.

**TABLE 2:** Summary of demographic and baseline transplant characteristics by treatment

Parameter^*a*^	Value		
	Placebo (*n* = 21)	Total active CSJ148 (*n* = 65)	Total active and placebo (*n* = 86)
Age (yr)			
Median	44.0	56.0	55.0
Range	22, 67	22, 76	22, 76
Sex [no. (%)]			
Male	15 (71.4)	40 (61.5)	55 (64.0)
Female	6 (28.6)	25 (38.5)	31 (36.0)
Race [no. (%)]			
White	7 (33.3)	36 (55.4)	43 (50.0)
Asian	14 (66.7)	25 (38.5)	39 (45.3)
Other	0 (0.0)	4 (6.2)	4 (4.7)
BMI^*b*^ (kg/m^2^)			
Median	23.7	25.7	24.8
Range	19, 29	19, 33	19, 33
Donor type [no. (%)]			
Not related	7 (33.3)	36 (55.4)	43 (50.0)
Related	14 (66.7)	29 (44.6)	43 (50.0)
Stem cell source [no. (%)]			
Bone marrow	1 (4.8)	11 (16.9)	12 (14.0)
Peripheral blood	20 (95.2)	52 (80.0)	72 (83.7)
Cord blood	0 (0.0)	2 (3.1)	2 (2.3)
Transplant type [no. (%)]			
Match	15 (71.4)	52 (80.0)	67 (77.9)
Haploidentical	5 (23.8)	10 (15.4)	15 (17.4)
Other	1 (4.8)	3 (4.6)	4 (4.7)
Donor HCMV seropositive [no. (%)]			
No	5 (23.8)	22 (33.8)	27 (31.4)
Yes	15 (71.4)	41 (63.1)	56 (65.1)
Unknown	1 (4.8)	2 (3.1)	3 (3.5)
Conditioning regimen [no. (%)]			
Nonmyeloablative	11 (52.4)	39 (60.0)	50 (58.1)
Myeloablative	10 (47.6)	26 (40.0)	36 (41.9)
T cell depletion [no. (%)]			
No	15 (71.4)	61 (93.8)	76 (88.4)
Yes	6 (28.6)	4 (6.2)	10 (11.6)

a Analysis set, all patients.

b BMI, body mass index.

### Efficacy.

Efficacy was evaluated by monitoring clinical outcomes and HCMV loads. The primary endpoint was the proportion of patients who required preemptive anti-HCMV therapy or treatment for HCMV disease. In the full analysis set, 37% of patients who received CSJ148 and 43% of those receiving placebo required preemptive therapy. The Bayesian model using a historical placebo prior shows a reduction of approximately 11% (risk ratio, 0.89; 90% confidence interval [CI], 0.61 to 1.31). The estimated probability that CSJ148 therapy decreases the need for preemptive therapy compared to placebo was 69%. Similar trends were observed in all analysis populations examined (Table 3).

**TABLE 3:** Summary of the posterior distribution of the proportion of patients that required preemptive anti-HCMV therapy up to visit day 99

Set	CSJ148 [no. (%)]	Placebo [no. (%)]	Estimated CSJ148/placebo median risk ratio (90% CI)	Posterior probability of risk ratio <1
Full analysis	24/65 (36.9)	9/21 (42.9)	0.891 (0.606,1.305)	0.694
Combined PD	23/47 (48.9)	9/17 (52.9)	0.926 (0.615, 1.524)	0.611
Combined modified PD	17/41 (41.5)	8/16 (50.0)	0.829 (0.515, 1.449)	0.722

**FIG 1:**
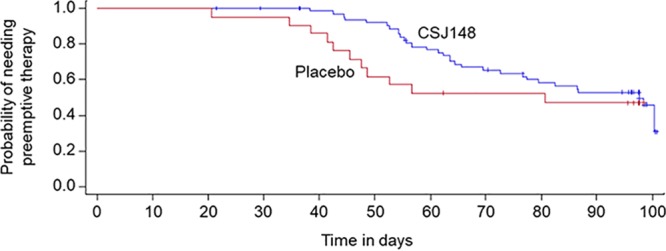
Cumulative survival free of the need to start preemptive therapy for HCMV. Patients treated with CSJ148 and those receiving placebo are shown. The time to start of preemptive therapy was defined as the number of days between the initial dose of study drug and (i) the start of preemptive therapy, (ii) the development of HCMV disease, or (iii) death due to HCMV disease. The log rank *P* value was 0.282 for comparison of the two curves.

The median viral load at the initiation of preemptive therapy for CSJ148-treated patients was 1,914 copies/ml (range, 120 to 45,430 copies/ml) compared with 5,555 copies/ml (range, 875 to 15,620 copies/ml) for placebo-treated patients. One subject who received CSJ148 had HCMV loads below the limit of quantification. For patients who had HCMV loads of >1,000 copies/ml at the initiation of the first course of preemptive therapy (*n* = 29), 13/20 (65%) CSJ148-treated patients and 6/9 (67%) placebo-treated patients achieved viral loads below the lower limit of quantification before the end of treatment on day 99. The median time to achieve a viral load below the lower limit of quantification after the first course of preemptive therapy was shorter in CSJ148-treated (median, 20 days; range, 5 to 36 days) than in placebo-treated (median, 30 days; range, 15 to 52 days) patients. The median time to start of therapy was 97.6 days (95% CI, 76.5 days [upper limit not calculated]) and 80.6 days (95% CI, 45.5 days [upper limit not calculated]) among CSJ148-treated (*n* = 24) and placebo-treated (*n* = 9) patients, respectively (log rank *P* value, 0.282) (Fig. 1).

Although not statistically significant (*P* = 0.335), the median duration of preemptive therapy tended to be shorter in CSJ148-treated (35 days) than in placebo-treated (39 days) patients. The numbers of courses of preemptive therapy required were 2 and 3 for CSJ148- and placebo-treated patients, respectively.

Eight patients developed HCMV disease (including the cases occurring after day 99), with more occurring in CSJ148-treated patients (7/65; 11%) than in placebo-treated patients (1/21; 5%). All the cases, except one diagnosed in a CSJ148-treated patient, had tissue evidence of HCMV disease based on histology or detection of viral DNA in the target organs. Five of the patients receiving CSJ148 developed HCMV disease prior to the end-of-treatment visit on day 99; all five had developed GVHD prior to the diagnosis of HCMV disease. During the follow-up period, two CSJ148-treated and one placebo-treated patients developed HCMV disease; none had prior GVHD.

Exploratory *post hoc* analyses using the combined PD analysis set were performed to determine if the lack of efficacy could be explained in part by confounding factors. Although this was a *post hoc* assessment and the sample size was too small to draw definitive conclusions, potentially relevant associations of three baseline factors and one postenrollment factor with the primary outcome were noted (based on a Cochran-Mantel-Hazard [CMH] test; *P* ≤ 0.300) (Table 4). The baseline factors were receiving a myeloablative versus a nonmyeloablative conditioning regimen (*P* = 0.146), receiving a mismatched versus a matched transplant (*P* = 0.009), and receiving stem cells from an unrelated donor versus a related donor (*P* = 0.039). The postenrollment factor was development of acute GVHD (*P* = 0.001). Other factors examined but found to be unassociated with the primary outcome (*P* > 0.300) included donor HCMV serostatus, T cell depletion of the graft, and development of chronic GVHD. The source of the stem cells was also considered but not examined because no placebo-treated patient received cord blood.

**TABLE 4:** Summary of associations between potential confounding factors and primary endpoint^*a*^

Category	Subcategory	Value^*b*^			*P* value^*c*^
		CSJ148 (cohorts 1 and 2) [*n*/*N* (%, 90% CI)]	Placebo (cohort 2) [*n*/*N* (%, 90% CI)]	Total [*n*/*N* (%)]	
Postenrollment factors					
Acute GVHD	Yes	17/24 (71, 52–85)	4/5 (80, 34–99)	21/29 (72.4)	0.001
	No	6/23 (26, 12–45)	5/12 (42, 18–68)	11/35 (31.4)	
Chronic GVHD	Yes	3/9 (33, 10–66)	2/4 (50, 10–90)	5/13 (38.5)	0.351
	No	20/38 (53, 38–67)	7/13 (54, 29–78)	27/51 (52.9)	
Baseline factors					
Age	>40 yr	20/41 (49, 35–63)	5/10 (50, 22–78)	25/51 (49.0)	0.818
	≤40 yr	3/6 (50, 15–85)	4/7 (57, 23–87)	7/13 (53.8)	
	>55 yr	11/24 (46, 28–64)	2/5 (40, 8–81)	13/29 (44.8)	0.483
	≤55 yr	12/23 (52, 34–70)	7/12 (58, 32–82)	19/35 (54.3)	
Conditioning regimen	Myeloablative	14/20 (70, 49–86)	4/10 (40, 15–70)	18/30 (60.0)	0.146
	Nonmyeloablative	9/27 (33, 19–51)	5/7 (71, 34–95)	14/34 (41.2)	
Donor HCMV seropositive	No	9/17 (53, 31–74)	3/5 (60, 19–92)	12/22 (54.5)	0.579
	Yes	13/29 (45, 29–62)	6/11 (55, 27–80)	19/40 (47.5)	
Donor type	Not related	16/23 (70, 50–85)	2/5 (40, 8–81)	18/28 (64.3)	0.039
	Related	7/24 (29, 15–48)	7/12 (58, 32–82)	14/36 (38.9)	
Stem cell source	Cord blood	0/1 (0.0)	0	0/1 (0.0)	0.328
	Peripheral blood or bone marrow	23/46 (50, 37–63)	9/17 (53, 31–74)	32/63 (50.8)	
T cell depletion	Yes	0/2 (0.0)	3/4 (75, 25–99)	3/6 (50.0)	0.933
	No	23/45 (51, 38–64)	6/13 (46, 22–71)	29/58 (50.0)	
Transplant type	Nonmatch	7/10 (70, 39–91)	5/5 (100, 55–100)	12/15 (80.0)	0.009
	Match	16/37 (43, 29–58)	4/12 (33, 12–61)	20/49 (40.8)	

a Analysis set for the table, combined cohort 1 and 2 PD analysis set.

b *n*, number of subjects who reached the primary endpoint; *N*, number of patients with or without the given factor.

c *P* value based on CMH test stratified by treatment group.

Three of the four potentially confounding factors showed a trend toward association with the treatment group. Proportionally more CSJ148-treated patients received a transplant from an unrelated donor (*P* = 0.254) and developed acute GVHD (*P* = 0.160). Proportionally more patients who received placebo received a myeloablative conditioning regimen (*P* = 0.272) (Table 5).

**TABLE 5:** Summary of associations between potential confounding factors and treatment

Category	Subcategory	Value [*n*/*N*^*a*^ (%)]		*P* value^*b*^
		CSJ148 (cohorts 1 and 2)	Placebo (cohort 2)	
Postenrollment factors				
Acute GVHD	Yes	24/47 (51.1)	5/17 (29.4)	0.160
	No	23/47 (48.9)	12/17 (70.6)	
Chronic GVHD	Yes	9/47 (19.1)	4/17 (23.5)	0.732
	No	38/47 (80.9)	13/17 (76.5)	
Baseline factors				
Age	>40 yr	41/47 (87.2)	10/17 (58.8)	0.030
	≤40 yr	6/47 (12.8)	7/17 (41.2)	
	>55 yr	24/47 (51.1)	5/17 (29.4)	0.160
	≤55 yr	23/47 (48.9)	12/17 (70.6)	
Conditioning regimen	Myeloablative	20/47 (42.6)	10/17 (58.8)	0.272
	Nonmyeloablative	27/47 (57.4)	7/17 (41.2)	
Donor HCMV seropositive	No	17/46 (37.0)	5/16 (31.3)	0.769
	Yes	29/46 (63.0)	11/16 (68.8)	
Donor type	Not related	23/47 (48.9)	5/17 (29.4)	0.254
	Related	24/47 (51.1)	12/17 (70.6)	
Stem cell source	Cord blood	1/47 (2.1)	0/17	1.000
	Peripheral blood or bone marrow	46/47 (97.9)	17/17 (100.0)	
T cell depletion	Yes	2/47 (4.3)	4/17 (23.5)	0.038
	No	45/47 (95.7)	13/17 (76.5)	
Transplant type	Nonmatch	10/47 (21.3)	5/17 (29.4)	0.517
	Match	37/47 (78.7)	12/17 (70.6)	

a *n*, number of subjects with or without the given factor; *N*, number of patients in each treatment group.

b Based on 2-sided Fisher’s exact test.

### Pharmacokinetics.

Following intravenous (i.v.) administration of CSJ148, LJP538, and LJP539, serum concentrations of both antibodies increased rapidly and reached the maximum concentration around the end of infusion. Serum concentrations decreased quickly after the infusion ended, with a rapid distribution phase followed by a much slower terminal elimination phase. Concentrations of both LJP538 and LJP539 increased with each subsequent dose. The mean accumulation ratios from first to last dose were 1.83 and 2.11 for LJP538 and LJP539, respectively. The mean terminal elimination half-lives after the last dose were estimated to be 19.7 and 23.8 days for LJP538 and LJP539, respectively. The absence of accelerated clearance during the long terminal elimination phase suggests that significant levels of anti-drug antibodies had not developed during the study. Overall, the pharmacokinetic (PK) characteristics of both LJP538 and LJP539 were typical of other human IgG1 antibodies and similar to that seen in healthy volunteers.

In the 50 patients who received all four doses of CSJ148, the trough concentrations for LJP538 and LJP539 were above the targeted efficacious concentrations (7.4 and 0.74 μg/ml for LJP538 and LJP539, respectively). There was no relationship between trough concentrations and the need for preemptive anti-HCMV therapy (data not shown).

### Safety.

Significant morbidity and mortality are seen in patients undergoing HCT. Consistent with this, all the patients (100%) in the safety analysis set reported at least one treatment-emergent adverse event (see Table S1 in the supplemental material**)**. There were 22 deaths during the study. Over 80% of the patients receiving placebo or CSJ148 developed at least one adverse event of grade 3 or higher severity, and the majority developed at least one serious adverse event (46/65 [71%] for CSJ148 and 15/21 [71%] for placebo). The most frequently reported serious adverse events—acute GVHD, febrile neutropenia, acute myeloid leukemia relapse, pneumonia, sepsis, and acute kidney injury—are well-known direct complications of allogeneic HCT or the subsequent increased susceptibility to disease.

The most common (>50% incidence) treatment-emergent adverse events were nausea (70% of total patients), diarrhea (65%), vomiting (61%), stomatitis (59%), and pyrexia (57%). The incidences of grade 3 or higher adverse events were similar among CSJ148- and placebo-treated patients. Based on an absolute difference of at least 10%, however, eight adverse events occurred more frequently in CSJ148-treated patients (diarrhea, hypertension, dizziness, hemorrhoids, arthralgia, and noncardiac chest pain) and three occurred more frequently in placebo-treated patients (stomatitis, upper abdominal pain, and neutropenia). Only two adverse events were assessed as possibly related to the study drug (fever and thrombocytopenia); both occurred in patients receiving placebo. The adverse events occurring in more than 10% of either treatment group are listed in Table S1.

Fifty-eight of 65 (89.2%) CSJ148-treated and 17 of 21 (81.0%) placebo-treated patients had at least one grade 3 or higher adverse event; the most common were febrile neutropenia (30.2% of total patients), stomatitis (18.6%), and hypertension (15.1%) (Table 6). In general, the incidences of all adverse events were similar for CSJ148- and placebo-treated patients. Based on an absolute difference of at least 10%, stomatitis and acute graft-versus-host disease occurred more frequently in CSJ148-treated patients, and no adverse event occurred more frequently in placebo-treated patients. As expected in the population studied, all the patients experienced hematologic abnormalities. Among grade 4 hematologic adverse events with differences of at least 10%, patients in the CSJ148-treated group had more frequent decreased platelet counts (73.8% versus 61.9% in the placebo-treated group) and decreased neutrophil count (53.8% versus 33.3% in the placebo-treated group), while grade 4 reduction in lymphocyte count was more common in placebo-treated patients (100%) than CSJ148-treated patients (84.6%) (see Table S2 in the supplemental material). Most clinical-chemistry abnormalities were assessed as being part of the underlying disease process and transplantation, related to a subsequent adverse event, or considered not clinically relevant.

**TABLE 6:** Incidence of grade 3 or higher adverse events (>10%) by preferred term

Preferred term	Value [*n* (%)]		
	Placebo (*n* = 21)	Total active CSJ148 (*n* = 65)	Total active and placebo (*n* = 86)
Febrile neutropenia	5 (23.8)	21 (32.3)	26 (30.2)
Stomatitis	2 (9.5)	14 (21.5)	16 (18.6)
Hypertension	2 (9.5)	11 (16.9)	13 (15.1)
Diarrhea	3 (14.3)	8 (12.3)	11 (12.8)
Thrombocytopenia	3 (14.3)	8 (12.3)	11 (12.8)
Nausea	2 (9.5)	8 (12.3)	10 (11.6)
Acute GVHD	1 (4.8)	10 (15.4)	11 (12.8)
Neutropenia	2 (9.5)	7 (10.8)	9 (10.5)
Acute kidney injury	1 (4.8)	7 (10.8)	8 (9.3)
Anemia	3 (14.3)	5 (7.7)	8 (9.3)
Pneumonia	1 (4.8)	7 (10.8)	8 (9.3)

Chronic GVHD (any grade) developed in 15% (10/65) of CSJ148-treated patients compared with 19% (4/21) of placebo-treated patients (*P* = 0.738). Acute GVHD developed in 43% (28/65) of CSJ148-treated patients compared with 29% (6/21) of placebo-treated patients (*P* = 0.308). There were 10 (15%) CSJ148-treated patients and 1 (5%) placebo-treated patient with severe (grade 3 or higher) acute GVHD (including intestine, liver, and skin). There were three (4.6%) CSJ148-treated patients who experienced severe chronic GVHD, and none in the placebo arm.

Twenty-two patients died during the study: 19/65 (29%) patients treated with CSJ148 and 3/21 (14%) who received placebo. The median times to death following the first dose of CSJ148 or placebo were 159 and 158 days, respectively. Of the 19 deaths in the CSJ148-treated patients, 4 (6.2%) occurred during the treatment period (up to day 99), 8 (12.3%) during follow-up (days 100 to 183), and 7 (10.8%) after the end-of-study visit (after day 184). All deaths in placebo-treated patients occurred after the end-of-treatment visit, with two (9.5%) deaths during the follow-up period and one (4.8%) after the end-of-study visit. No patient deaths were suspected to be related to the study drug, and all causes of death were consistent with those expected after allogeneic HCT (see Table S3 in the supplemental material).

Exploratory *post hoc* analyses were performed among all patients in the safety set to determine if the death rates could be explained in part by confounding factors. Potentially relevant associations (based on a Cochran-Mantel-Hazard test *P* value of ≤0.3) were noted between mortality and age (>55 versus ≤55 years of age; *P* < 0.3), transplant type (mismatched versus matched; *P* < 0.01), and conditioning regimen (myeloablative versus nonmyeloablative; *P* = 0.01). Of these, only age >55 versus ≤55 years (*P* < 0.10) showed an association with the treatment group based on a Fisher’s exact test *P* value of ≤0.3; proportionally more patients >55 years of age received CSJ148 (52.3%) than placebo (28.6%). Two additional factors reported to be associated with mortality (11) also showed an association with the treatment group: age >40 versus ≤40 years (*P* < 0.01) and unrelated versus related donor type (*P* < 0.3). Proportionally more CSJ148-treated patients were >40 years of age (87.7% versus 51.7%) or received a transplant from an unrelated donor (55.4% versus 33.3%).

It was possible that CSJ148 might enhance tissue damage via antibody-dependent cell-mediated cytotoxicity in the presence of HCMV replication. To investigate this, a temporal analysis of deaths in relation to acute GVHD, clinically relevant HCMV infection, HCMV disease, and use of rescue anti-HCMV therapy was conducted among the CSJ148-treated patients. Ten deaths occurred in patients without evidence of clinically relevant HCMV infection or disease. Seven of these 10 patients were >55 years old. No clear temporal correlation with CSJ148 administration was identified, and only three deaths occurred during the study treatment period. Nine deaths occurred in patients with evidence of clinically relevant HCMV infection (*n* = 4) or disease (*n* = 5). Six of these patients were >55 years old. All nine of the patients received rescue anti-HCMV therapy, and all but one had controlled HCMV infections (viral load < 1,000 copies/ml) prior to death. The one exception was a patient with a viral load of 2,948 copies/ml in the week preceding death, down from a peak value of >1 million copies/ml after receiving anti-HCMV therapy. This patient died during the treatment period from atypical pneumonia after being diagnosed with GVHD and HCMV colitis.

All the subjects with HCMV disease and two subjects with HCMV infection had GVHD diagnosed prior to or concurrently with HCMV. GVHD is a known risk factor for HCMV disease. Taken together, these results indicate it was unlikely that CSJ148 exacerbated end organ disease.

## DISCUSSION

In this randomized phase 2 trial, in CSJ148-treated patients, the primary endpoint of preventing HCMV infection or reactivation during the first 98 days after allogeneic HCT was not met. The estimated probability that CSJ148 decreases the need for preemptive therapy compared to placebo was 69%. The risk ratio of 0.89 (90% CI, 0.61 to 1.31) was not statistically significant, and the wide 90% CI of the risk ratio suggests significant variability in the treatment effect. Secondary-efficacy results showed trends for a later time to the start of preemptive therapy, a shorter duration of preemptive therapy, and the need for fewer preemptive treatment courses. Although a higher rate of CSJ148-treated patients (11%) than placebo-treated patients (5%) developed HCMV disease, the difference was not statistically significant, and the small number of events precludes any definitive conclusions.

In the general allogeneic HCT population, the development of acute or chronic GVHD and certain baseline transplant characteristics (HCMV-seropositive donor, myeloablative conditioning regimen, unrelated donor, mismatched donor, cord blood as the source of the stem cells, and T cell depletion) are associated with a higher degree of immunosuppression and are considered to increase the risk of HCMV reactivation (12, 13). Additional exploratory *post hoc* analyses to determine if the lack of efficacy could be explained in part by confounding factors showed that a potentially disproportionate number of CSJ148-treated patients (51%) developed acute GVHD compared with those who received placebo (29%), and the development of acute GVHD was associated with a greater risk of requiring preemptive therapy for HCMV (71% versus 26% if acute GVHD did not occur) in CSJ148-treated patients. CSJ148-treated patients (55%) were more likely to have received a graft from an unrelated donor than those who received placebo (33%), and unrelated grafts appeared to also be associated with a greater risk of requiring preemptive therapy for HCMV (70% versus 30% from related donors) in CSJ148-treated patients. Because of the small trial size, imbalances in relevant confounders between treatment arms may have occurred by chance. In this trial, it appears that proportionally more patients treated with CSJ148 may have had more potentially confounding attributes. It is possible that a combination of confounders could have influenced the outcomes reported here. In addition, failure to demonstrate efficacy with CSJ148 does not preclude the possibility that other antibody-mediated therapies will not work.

In transplant patients and those with HIV disease and acquired immunodeficiency, severe HCMV disease occurs almost exclusively in patients with profound T cell immune deficiency (14). T cell responses correlate with protection and recovery from HCMV disease. Infusion of HCMV-specific T cells has been used to restore cellular immunity and to offer protection against HCMV disease (15, 16). Conversely, anti-HCMV antibodies show only modest activity for preventing HCMV infection in transplant patients. HCMV hyperimmune globulin is approved for the prevention of HCMV infection and disease in solid organ transplantation, but not in HCT (17). Monoclonal IgG antibodies show only modest effects in preventing HCMV infection after kidney transplantation, but not after HCT (18). Together, these data suggest that the failure to meet the primary endpoint could be due to an inability of CSJ148 to overcome the severe deficiencies in T cell immunity seen among HCT patients in this study. However, CSJ148 did appear to have anti-HCMV activity, and CSJ148 may be effective in lower-risk HCT populations or in combination with anti-HCMV T cell immunity enhancers.

In contrast to preventing HCMV reactivation in transplant patients, prevention of primary infection *in utero* might be possible with CSJ148. In HCMV congenital disease, a predominant protective role of high-avidity IgG has been described. Preexisting IgG reduced fetal transmission by 69% in pregnant women (19), and an anti-gH/gL monoclonal IgG was protective in a congenital guinea pig model (20). HCMV hyperimmune globulin resulted in a statistically insignificant reduction in HCMV transmission to the fetus compared with placebo (44% versus 30%) in 124 pregnant women with a primary HCMV infection. This study, however, was designed to detect a 50% reduction in fetal HCMV transmission, and the 33% relative risk reduction that was noted (*P* = 0.13) may be clinically relevant (21).

CSJ148 was well tolerated in the HCT patient population. Although there were multiple serious adverse events reported in this population, the majority of events were consistent with those expected in patients undergoing HCT. The incidences of adverse events and serious adverse events were similar between the treatment groups.

GVHD, a known complication of allogeneic HCT, was observed in higher proportion in the CSJ148-treated patients. The reason for the higher rate in CSJ148-treated patients is not clear, although these patients were older, and older age is a risk factor for acute GVHD (22–26). Other imbalances in baseline characteristics may have also contributed to the higher proportion of graft-versus-host disease observed in CSJ148-treated patients. CSJ148-treated subjects had a lower proportion of T cell-depleted grafts than placebo-treated subjects (6.2% and 28.6%, respectively) and a higher proportion of nonrelated transplants (55.4% and 33.3%, respectively); both are risk factors for GVHD (25, 27). Pooled immunoglobulin and monoclonal antibody preparations are not known to cause or promote GVHD.

Overall mortality in the study population was consistent with the reported mortality in HCT patients (29% to 66%) (11). All causes of death were consistent with those expected after HCT, and none were suspected to be related to the study drug by the investigators. A plausible direct causal relationship between CSJ148 treatment and increased mortality was not identified, and the majority of deaths occurred after the treatment period (during follow-up or after study completion). Although the sample size is too small to draw definitive conclusions, *post hoc* analysis identified older age as a potential confounder for mortality. Other factors potentially associated with mortality examined included age (>40 versus ≤40 years of age), donor type (unrelated versus related), and presence of acute GVHD. Although none of them showed a trend toward a potentially relevant association, all have been reported by others to increase the risk of death (11, 28). Taken together, the data suggest a higher mortality rate among CSJ148-treated patients than among placebo-treated patients being driven by these patients having a higher mortality risk at baseline.

In conclusion, consistent with the experience in healthy volunteers, CSJ148 was well tolerated and appears to have anti-HCMV activity. However, treatment did not prevent clinically significant HCMV reactivation in HCMV-seropositive allogeneic HCT recipients.

## MATERIALS AND METHODS

### Study design.

This phase 2 multicenter study was designed to evaluate the efficacy, safety, and tolerability of CSJ148 in patients undergoing allogeneic HCT. The study comprised a screening period, a baseline visit, a treatment period of approximately 3 months (days 1 to 99), an end-of-therapy visit, a follow-up period (days 100 to 183), and an end-of-study visit approximately 3.5 months after the final dose.

The study consisted of two cohorts. Cohort 1 (single arm and noncontrolled) was designed to evaluate the PK of CSJ148 in transplant recipients. Six patients were enrolled in cohort 1; all received i.v. CSJ148 every 28 days for the duration of the treatment period (99 days). Cohort 2 was randomized, double blinded, and placebo controlled. The 80 patients enrolled in cohort 2 were randomized to CSJ148 or placebo in a 3:1 ratio, with dosing every 28 days (days 1, 29, 57, and 85). For all patients who received CSJ148 in either cohort, the doses consisted of 50 mg/kg of body weight LJP538 infused i.v. over 2 h and 5 mg/kg LJP539 infused over 0.2 h. The Institutional Review Board (IRB) or ethics committees approved the study protocol and all amendments. All subjects provided written informed consent.

### Patients.

Eligible patients were males and females ≥18 years of age undergoing allogeneic HCT of all types (myeloablative or reduced-intensity conditioning using related, unrelated, or haploidentical donors) and for any indication. All the patients were required to be HCMV seropositive before transplantation. Key exclusion criteria included HCMV-related organ disease within 6 months of enrollment, detectable HCMV infection (determined by positive pp65 antigenemia or plasma HCMV DNA PCR at local laboratories) within 14 days prior to enrollment, or use of any medications with anti-HCMV activity within 30 days prior to enrollment. Patients were also excluded if they had a history of hypersensitivity to monoclonal antibodies, impaired renal function requiring dialysis, severe liver disease, or any surgical or medical condition that might increase the risk for thrombotic events (including cryoglobulinemia, monoclonal gammopathies, and severe hypertriglyceridemia).

### Trial assessments.

Efficacy was assessed through monitoring of both HCMV loads and clinical outcomes, including the development of HCMV disease and the use of rescue medication to treat HCMV (based on the need for preemptive therapy or therapy for HCMV disease). The primary efficacy endpoint was the proportion of patients who required preemptive anti-HCMV therapy based on a plasma HCMV DNA of ≥1,000 copies/ml (based on central-laboratory results) or who developed HCMV disease within 99 days after the start of dosing. HCMV loads were monitored weekly during the treatment period. Secondary endpoints included the time to start and duration of preemptive therapy, the number of preemptive therapy courses required, and the proportion of patients developing HCMV disease, as defined in published criteria (29) and detailed in the supplemental material. Safety assessments consisted of collecting all adverse events and regular monitoring of hematology and blood and urine chemistry. Blood samples for PK and CSJ148 immunogenicity (anti-drug antibodies) were collected throughout the study and analyzed as previously described (10).

### Statistical methods.

This study was designed to determine if CSJ148 could prevent HCMV replication after HCT and therefore reduce the proportion of patients requiring preemptive anti-HCMV therapy or developing HCMV disease. The analysis sets are described in Table 1, with exclusions outlined in the supplemental material.

The safety (or full analysis) set included all patients who received at least one dose of the study drug. The PK analysis set included patients in the full analysis set with available PK data and no protocol deviations that could influence the results. The PD analysis set included patients from cohort 2 in the full analysis set with available PD data and no protocol deviations that could impact the results, such as missing any dose of the study drug, use of medications with anti-HCMV activity when the primary endpoint was not met, and non-HCMV-related death. Additional PD analyses included all the patients in cohorts 1 and 2 who met the criteria for the first PD analysis set and patients who received anti-HCMV medications who were otherwise eligible for the PD analysis sets (modified PD).

The primary endpoint was summarized with descriptive statistics by cohort and treatment arm. For cohort 2, the primary endpoint was modeled with the binomial distribution for the event probabilities for the treatment arm (pT) and the control arm (pC). The pT was given by a noninformative beta prior distribution with parameters 1/3 and 1/3. A Bayesian analysis with an informative prior for the control arm was applied to the full analysis set. The pC was given by an informative prior (based on a meta-analysis conducted on historical studies with similar designs). The informative prior was a mixture of three beta distributions with parameters equal to 19.49 and 28.80 (first beta component), 3.88 and 5.11 (second beta component), and 1 and 1 (third beta component). The mixture weights were 0.64, 0.31, and 0.05, respectively. Based on these priors and the observed primary outcome of this study, posterior distributions for pT and pC were computed. These posterior distributions were summarized by the posterior medians and standard deviations. Ninety percent credible intervals were also provided.

The posterior distribution for pT/pC (the risk ratio) was computed and summarized in a similar fashion. Additionally, posterior probabilities (confidence levels) that the risk ratio was <1 and <0.5 were computed.

Exploratory *post hoc* subgroup analyses to assess the effects of potential confounding factors based on baseline and postenrollment factors on patients requiring preemptive HCMV therapy were performed as needed. Tests of associations between potential confounding factors and primary outcomes adjusting for treatment were performed using a CMH test, while Fisher’s exact test was used to test for associations between potential confounding factors and treatment. Since the small sample size limited the power to identify associations, we considered *P* values of <0.30 as potential signals of association, with the caveat of a high false-positive rate (3 in 10 chances of falsely rejecting the null hypothesis of no association).

Safety endpoints were summarized using descriptive statistics. Secondary and exploratory analyses included time to start and duration of preemptive therapy, number of preemptive therapy courses required, HCMV load at the start of preemptive therapy, and the proportion of patients developing HCMV disease.

## Supplementary Material

Supplemental file 1
