# Supplementary material for: Phase 2 Study of Anti-Human Cytomegalovirus Monoclonal Antibodies for Prophylaxis in Hematopoietic Cell Transplantation
Source: Antimicrob Agents Chemother. 2020 Mar 24;64(4):e02467-19. doi: 10.1128/AAC.02467-19 (PMC7179282; doi:10.1128/AAC.02467-19)
Supplement: Supplemental file 1 [file AAC.02467-19-s0001.pdf]

Supplemental Information

Assessment of human cytomegalovirus (HCMV) disease

HCMV disease was defined according to published criteria. For pneumonia, central nervous system (CNS) disease, gastrointestinal disease, hepatitis, nephritis, cystitis, myocarditis, pancreatitis, and disease in other organs, definite tissue-invasive disease requires the correct clinical syndrome combined with the detection of HCMV in tissue samples (or in bronchoalveolar lavage fluid for pneumonia) by virus isolation, immunohistochemical analysis, in situ hybridization, or conventional histologic features. Detection of HCMV by polymerase chain reaction (PCR) alone is not sufficient. For CNS disease, detection of HCMV in cerebrospinal fluid (CSF) samples by culture or PCR is sufficient. HCMV viral syndrome requires fever (oral temperature >38°C) for 2 or more days within a 4-day period, neutropenia or thrombocytopenia, and the detection of HCMV in the blood by culture or the detection of antigen, DNA, or RNA. Human herpesvirus 6 infection needs to be excluded. HCMV-associated graft failure requires severe pancytopenia, bone marrow hypoplasia, and the detection of HCMV by culture in bone marrow. Graft-versus-host disease, relapse, and human herpesvirus 6 infection all need to be excluded. Probable disease requires the correct clinical syndrome but the detection of HCMV cannot be confirmed as outlined above. For retinitis, typical HCMV lesions must be confirmed by an ophthalmologist; detection of HCMV is not required.

Statistical methods

Kaplan-Meier plots and the log-rank test were used to assess the time to start of preemptive therapy between the treatment groups. The duration (in days) of preemptive therapy was first averaged across administrations per patient and summarized across patients and treatment group. The difference between treatment groups was assessed using the Hodges-Lehman location test. Descriptive summaries were performed on the number of times preemptive therapy is required and on the proportion of patients developing HCMV disease. Safety endpoints were summarized using descriptive statistics. Exploratory analyses including Cochran-Mantel Hazard test to assess the effect of potential confounding factors on mortality rates adjusting for treatment were

performed on the safety analysis set. Fisher's exact test was also performed to test the association between potential confounding factors and treatment group.

Exclusions from analysis sets. Eighty-six patients were randomized, received at least one dose of CSJ148 and were included in the safety analysis set and full analysis set. The combined (Cohort 1 and 2) pharmacodynamic (PD) analysis set included 64 patients, 22 patients (26%) were excluded. Among the 22 excluded patients, 14 (64%) patients were excluded for use of prohibited or rescue medications with anti-HCMV activity and did not meet the primary endpoint, 7 (32%) patients were excluded for study withdrawal or death prior to meeting the primary endpoint or Day 99 (whichever came first); and 1 (5%) patient who met the primary endpoint was excluded for missing a study drug dose prior to meeting the primary endpoint. The modified combined modified PD analysis set included 57 patients, 29 patients (34%) were excluded. Among the 29 excluded patients in the modified PD analysis set pooling Cohorts 1 and 2, 21 (72%) patients were excluded for use of prohibited or rescue medications with anti-HCMV activity, 7 (24%) were excluded for study withdrawal or death prior to meeting the primary endpoint or Day 99 (whichever came first); and 1 (3%) patient who met the primary endpoint was excluded for missing a study drug dose prior to meeting the endpoint.

Table S1: Number (percent) of patients in the analysis sets

Analysis set for this table: All patients

	Cohort 1	Cohort 2			
	**CSJ148 N=6 n (%)**	**CSJ148 N=59 n (%)**	**Placebo N=21 n (%)**	**Total CSJ148 N=65 n (%)**	**Total N=86 n (%)**
Patients randomized	6 (100.0)	59 (100.0)	21 (100.0)	65 (100.0)	86 (100.0)
Safety analysis and full analysis set	6 (100.0)	59 (100.0)	21 (100.0)	65 (100.0)	86 (100.0)
PK analysis set (CSJ148 only)	6 (100.0)	59 (100.0)	NA	65 (100.0)	65 (75.6)
PD analysis set (Cohort 2 only)	NA	42 (71.2)	17 (81.0)	42 (64.6)	59 (68.6)
Combined PD analysis set	5 (83.3)	42 (71.2)	17 (81.0)	47 (72.3)	64 (74.4)
Modified PD analysis set (Cohort 2 only)	NA	36 (61.0)	16 (76.2)	36 (55.4)	52 (60.5)
Combined modified PD analysis set	5 (83.3)	36 (61.0)	16 (76.2)	41 (63.1)	57(66.3)

NA = not applicable; PD = pharmacodynamic; PK = pharmacokinetic

Table S2: Incidence of adverse events >10% by preferred term - n (percent) of patients

Analysis set for this table: Safety analysis set

	Placebo N=21		Total CSJ148 N=65		Total N=86	
**Preferred term**	**n**	**(%)**	**n**	**(%)**	**n**	**(%)**
Nausea	14	(66.7)	46	(70.8)	60	(69.8)
Diarrhoea	12	(57.1)	44	(67.7)	56	(65.1)
Vomiting	14	(66.7)	38	(58.5)	52	(60.5)
Stomatitis	15	(71.4)	36	(55.4)	51	(59.3)
Pyrexia	11	(52.4)	38	(58.5)	49	(57.0)
Febrile neutropenia	11	(52.4)	29	(44.6)	40	(46.5)
Cough	9	(42.9)	25	(38.5)	34	(39.5)
Decreased appetite	7	(33.3)	27	(41.5)	34	(39.5)
Rash	7	(33.3)	24	(36.9)	31	(36.0)
Headache	6	(28.6)	24	(36.9)	30	(34.9)
Hypertension	5	(23.8)	23	(35.4)	28	(32.6)
Constipation	8	(38.1)	19	(29.2)	27	(31.4)
Fatigue	7	(33.3)	18	(27.7)	25	(29.1)
Hypokalaemia	5	(23.8)	20	(30.8)	25	(29.1)
Back pain	5	(23.8)	17	(26.2)	22	(25.6)
Pruritus	4	(19.0)	18	(27.7)	22	(25.6)
Abdominal pain	5	(23.8)	16	(24.6)	21	(24.4)
Insomnia	6	(28.6)	15	(23.1)	21	(24.4)
Acute graft versus host disease*	6	(19.0)	28	(23.1)	34	(22.1)
Chills	4	(19.0)	15	(23.1)	19	(22.1)
Dizziness	3	(14.3)	16	(24.6)	19	(22.1)
Dry eye	5	(23.8)	14	(21.5)	19	(22.1)
Dyspnoea	3	(14.3)	13	(20.0)	16	(18.6)
Haemorrhoids	2	(9.5)	13	(20.0)	15	(17.4)
Oropharyngeal pain	3	(14.3)	12	(18.5)	15	(17.4)
Dry mouth	3	(14.3)	11	(16.9)	14	(16.3)
Oedema peripheral	2	(9.5)	12	(18.5)	14	(16.3)
Arthralgia	1	(4.8)	12	(18.5)	13	(15.1)
Hypomagnesaemia	4	(19.0)	9	(13.8)	13	(15.1)
Hypotension	4	(19.0)	9	(13.8)	13	(15.1)
Upper respiratory tract infection	4	(19.0)	9	(13.8)	13	(15.1)
Abdominal pain upper	5	(23.8)	7	(10.8)	12	(14.0)
Neutropenia	5	(23.8)	7	(10.8)	12	(14.0)
Non-cardiac chest pain	1	(4.8)	11	(16.9)	12	(14.0)

Thrombocytopenia	3	(14.3)	9	(13.8)	12	(14.0)
Anaemia	4	(19.0)	7	(10.8)	11	(12.8)
Blood creatinine increased	3	(14.3)	8	(12.3)	11	(12.8)
Haematuria	1	(4.8)	10	(15.4)	11	(12.8)
Pleural effusion	1	(4.8)	10	(15.4)	11	(12.8)
Acute kidney injury	2	(9.5)	8	(12.3)	10	(11.6)
Bone pain	1	(4.8)	9	(13.8)	10	(11.6)
Chronic graft versus host disease*	4	(19.0)	10	(9.2)	10	(11.6)
Dry skin	4	(19.0)	6	(9.2)	10	(11.6)
Dyspepsia	3	(14.3)	7	(10.8)	10	(11.6)
Epistaxis	2	(9.5)	8	(12.3)	10	(11.6)
Oesophagitis	1	(4.8)	9	(13.8)	10	(11.6)
Pneumonia	1	(4.8)	9	(13.8)	10	(11.6)
Pollakiuria	2	(9.5)	8	(12.3)	10	(11.6)
Tachycardia	2	(9.5)	8	(12.3)	10	(11.6)
Abdominal distension	3	(14.3)	6	(9.2)	9	(10.5)
Alanine aminotransferase increased	3	(14.3)	6	(9.2)	9	(10.5)
Asthenia	2	(9.5)	7	(10.8)	9	(10.5)
Blood bilirubin increased	1	(4.8)	8	(12.3)	9	(10.5)
Folliculitis	2	(9.5)	7	(10.8)	9	(10.5)
Hyperglycaemia	1	(4.8)	8	(12.3)	9	(10.5)
Hypophosphataemia	3	(14.3)	6	(9.2)	9	(10.5)

*Related preferred terms pooled

Table S3: Overview of deaths

Analysis set for this table: Safety analysis set

Cause of death	Reason for HCT / Type of HCT	Relevant medical history	Study day: Death	Study day: Last dose of study drug
**CSJ148**				
Fungal pneumonia (mucormycosis)	Acute myeloid leukemia / unrelated, matched, non-myeloablative	71-year-old, atypical pneumonia	38	28
Progressive respiratory distress	Refractory myeloid leukemia / Matched, unrelated, non-myeloablative	71-year-old, pancytopenia, atrial hypertension and squamous nasal papilloma	38	28
Bacterial pneumonia, Epstein-Barr virus infection, respiratory insufficiency	Acute myeloid leukemia / Unrelated, matched, non-myeloablative	67-year-old, pneumonia fungal, empty sella syndrome, tobacco abuse	78	57
Atypical pneumonia	Myelodysplastic syndrome, acute myeloid leukemia / Related, matched, non-myeloablative	46-year-old, chronic hepatitis B, toxicoderma, edema of the limbs, febrile neutropenia	91	85
Acute hypoxic respiratory failure	Acute myeloid leukemia / Matched, unrelated, myeloablative	67-year-old, right atrial thrombus, elevated liver enzymes, diabetes mellitus, splenomegaly, anemia, neutropenia	100	58
Pneumonia, sepsis	Primary central nervous system lymphoma / Matched, related, myeloablative	64-year-old, hypertension	125	92
Relapse chronic myeloid leukemia in blast crisis	Chronic myeloid leukemia / Related, haploidentical, myeloablative	45-year-old, thrombosis (left vena jugularis), left eye retinal mass	141	85
Acute respiratory failure	Myelodysplastic syndrome / Related, haploidentical, non-myeloablative	71-year-old, steroid-induced diabetes, sleep apnea, hypertension	145	84
Septic shock	Chronic neutrophilic leukemia / Unrelated, matched, non-myeloablative	66-year-old, type 2 diabetes, myocardial infarction, hypercholesterolemia, hypertension	157	84
Sepsis	Myelodysplastic syndrome / Non-related, non-myeloablative	61-year-old, hypothyroidism, hypertension	164	84
Acute graft-versus-host disease skin, thrombotic thrombocytopenic purpura	Acute lymphoblastic leukemia / Related, haploidentical, non-myeloablative	47-year-old, fungal pneumonia, recurrent cytopenias, nephrolithiasis, hepatitis B virus carrier	166	85
Sepsis, relapsed acute myeloid leukemia	Acute myeloid leukemia / Matched, unrelated, non-myeloablative	59-year-old, type 2 diabetes mellitus, hypertension	159	8

Gastrointestinal bleeding	Natural killer T-cell lymphoma / Related, haploidentical, non-myeloablative	31-year-old, elevated transaminases, sinusitis	193	86
Acute graft-versus-host disease	Diffuse large B-cell lymphoma / Matched, unrelated, non-myeloablative	39-year-old, hypertension, seizure disorder, acute on chronic kidney disease, common variable immunodeficiency, hemolytic anemia	200	56
Acute myeloid leukemia relapse	Acute myeloid leukemia / Matched, related, non-myeloablative	68-year-old, hyperlipidemia, asthma, deep vein thrombosis	203	85
Gastrointestinal haemorrhage, acute renal failure	Acute myeloid leukemia / allogenic, related, non-myeloablative	57-year-old, chronic hepatitis B, fungal pneumonia, bacteremia	210	85
E. coli bacteremia, sepsis	Progressive non-Hodgkin lymphoma / Matched, unrelated, non-myeloablative	60-year-old, diabetes mellitus, urolithiasis, interstitial pneumonia, acute renal insufficiency, elevated aspartate and alanine aminotransaminases	213	84
Acute myeloid leukemia relapse	Acute myeloid leukemia / Matched, non-related, non-myeloablative	60-year-old, hypertension, systemic inflammatory response syndrome, and pulmonary embolism	221	86
Respiratory distress, relapsed chronic myelomonocytic leukemia	Chronic myelomonocytic leukemia / matched, unrelated, non-myeloablative	54-year-old, dyspnea on exertion, febrile neutropenia, hepatosplenomegaly, splenic infarction, chronic complex periodontitis	222	85
**Placebo**				
Febrile neutropenia	Acute myeloid leukemia / Related, haploidentical, non-myeloablative	22-year-old, fungal pneumonia, septic shock, human cytomegalovirus viremia	146	57
Intracranial haemorrhage	Acute myeloid leukemia / Related, haploidentical, non-myeloablative	55-year-old, Sjögren's disease, latent tuberculosis infection, hypertension, transient ischemic attack	158	58
Acute graft-versus-host disease gut and liver	Acute myeloid leukemia / Related, haploidentical, myeloablative	51-year-old, hypothyroidism	222	85

HCT = hematopoietic cell transplantation
